# Residual Stress Formation Mechanisms in Laser Powder Bed Fusion—A Numerical Evaluation

**DOI:** 10.3390/ma16062321

**Published:** 2023-03-14

**Authors:** Moritz Kaess, Martin Werz, Stefan Weihe

**Affiliations:** Materials Testing Institute (MPA), University of Stuttgart, Pfaffenwaldring 32, D-70569 Stuttgart, Germany; martin.werz@mpa.uni-stuttgart.de (M.W.);

**Keywords:** laser powder bed fusion, residual stress, distortion, numerical finite element simulation

## Abstract

Additive manufacturing methods, such as the laser powder bed fusion, do not need any special tool or casting mold. This enables the fast realization of complex and individual geometries with integrated functions. However, the local heat input during the manufacturing process often leads to residual stresses and distortion. This in turn causes poor quality, scrap parts or can even terminate a job prematurely if the powder recoating mechanism collides with a distorted part during the process. This study investigates the generation mechanisms of residual stresses and distortion during laser powder bed fusion (LPBF) of stainless steel 316L in order to reduce these effects and thus contribute to improved process safety and efficiency. Therefore, numerical investigations with a finite element model on the scale of a few melt tracks and layers serve to develop a detailed understanding of the mechanisms during production. The work includes an investigation of the build plate temperature, the laser power and speed and the layer thickness. The results show a strong dependency on the build plate preheating and energy per unit length. A higher build plate temperature and a reduction of the energy per unit length both lead to lower residual stresses.

## 1. Introduction

Laser powder bed fusion (LPBF) is an additive manufacturing technology, which uses a highly concentrated and very fast-moving laser heat source for selectively melting powder material. During the manufacturing process, high temperature gradients and transients occur locally which lead to the development of residual stresses and distortions in the manufactured parts [1,2].

**Figure 1:**
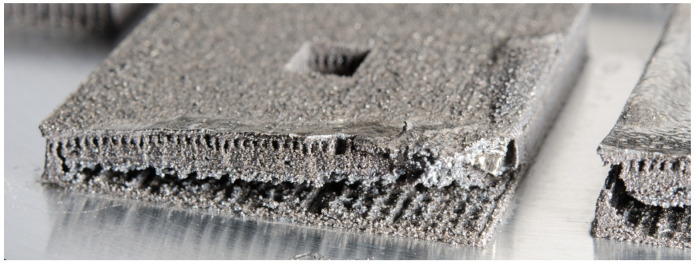
Distortions and delamination during manufacturing process leading to process failure.

Residual stresses in components can have a negative influence on the bearable loads in operation and thus can compromise the integrity of highly loaded parts because they superimpose with loading stresses. In order to guarantee for secure parts, manufacturers and users need to know these residual stresses. Additionally, distortions can lead to unwanted geometry deviations or even cause a collision of the powder recoater with the manufactured part during the manufacturing process, as shown in Figure 1. Manufacturer often optimize the process iteratively to avoid scrap parts and process abortions. However, these experimental studies are time-consuming and cost-intensive.

A numerical simulation of the LPBF manufacturing process is a method for predicting these residual stresses and distortions in parts before the actual manufacturing starts. A simulation allows for optimizing parameter configurations or even develop special process parameters for achieving desired part properties. The finite element method (FEM) is often used to simulate welding processes and related additive manufacturing processes. Different approaches are available depending on the calculation objective, such as thermal analysis, mechanical analysis and thermomechanically coupled analysis: If only the temperature distribution occurring during the manufacturing process is of interest, a heat transfer analysis [3] can be performed. If mechanical quantities, such as stresses, strains and deformations, are also to be calculated, various types of methods are available [4]: For example, the inherent strain method allows for a rapid prediction of residual stresses and distortion in whole components [4,5,6]. This approach only needs a mechanical simulation and uses a comparatively coarse discretization making it fast and feasible, but a numerical or experimental calibration is necessary. Temperature distribution is not simulated in this approach, which is a strong simplification. However, a detailed numerical analysis of the manufacturing process requires a high temporal and spatial resolution to precisely represent the laser spot diameter, laser movement as well as the layer thickness. For this purpose, the finite element method provides two approaches with different degrees of coupling. A “weakly-coupled thermomechanical analysis” [4], called “sequentially coupled thermal-stress analysis” in Abaqus, consists of two simulation steps. First, a transient temperature field is calculated in a heat transfer analysis, which serves as thermal input for a subsequent mechanical analysis. This method is suitable if the mechanical results do not influence the thermal behavior in the process to be simulated. It is assumed that in welding and additive manufacturing processes, such a feedback of mechanical quantities to thermal quantities can be neglected, making the sequentially coupled method suitable. The greatest computational effort is required for a “strongly-coupled thermomechanical analysis” [4], called “fully coupled thermal-stress analysis” in Abaqus. In this approach, the temperature field and the mechanical behavior are calculated simultaneously. This is useful if thermal and mechanical variables influence each other.

Numerous authors describe the use of finite element models to simulate the laser powder bed fusion manufacturing process and predict temperature fields, residual stresses and distortion. Often, small sections of components are simulated for detailed academic studies. Here it is common to model the manufacturing of one or more layers of geometries such as plates [7,8] and cuboids [9]. The solid build plate can be part of the model or represented by boundary conditions. Therefore, mechanical boundary conditions are regularly used to fix the build plate and thermal boundary conditions on various component surfaces take into account heat loss through thermal conduction, convection and thermal radiation. The layer-by-layer manufacturing process is usually implemented through a form of element activation [9,10]. A heat source for which there are different complex formulations usually models the laser radiation. On the one hand, heat sources with a uniformly distributed intensity [7,11] and, on the other hand, Gaussian distributed surface [12,13] and volume heat sources [8,14,15,16] are used. Goldak [17] compares different heat sources and describes their suitability for different welding processes, finally proposing an ideal double-ellipsoidal volume heat source for fusion welding processes. In addition, newer heat sources exist that have been developed to better account for laser coupling into the powder bed [18,19]. The coupling of the laser is quantitatively described by an efficiency present in the formulation of the heat source. To investigate this absorptivity, several authors examined the influence of the process parameters in LPBF and determined values between 30% and 70% depending on the welding regime [20,21,22]. Thermal and mechanical material behavior of metals is temperature dependent. In particular, for the calculation of distortion and residual stresses, it is usually necessary to use temperature-dependent material properties [7,10,11,14]. Some authors also describe a modeling of the transition from powder to molten and solid state [11,15]. The powder is usually modeled as a continuum by using adapted material properties such as reduced thermal conductivity [7,11].

These approaches contribute to gain a deeper understanding of the development of residual stresses in additive manufacturing processes. However, a high spatial and temporal resolution, which is necessary for a detailed modeling of the manufacturing process, is not feasible for the simulation of whole parts. In order to allow numerical simulations for realistic part dimensions with an appropriate computational effort, simplifications and modeling strategies considering only the relevant physical effects need to be chosen. Some authors describe multiscale approaches that combine different model resolutions or even different finite element approaches [5,6,23,24] in order to consider the process details on the one hand and to be able to calculate large components on the other hand.

In addition, related additive manufacturing processes such as directed energy deposition (DED) [25] and wire arc additive manufacturing (WAAM) [26] can also be described using the simulation approaches presented above. Simulation objective is also a prediction of the development of residual stresses dependent on the production parameters. Here, simulations show an influence of the scanning strategy as well as the preheating and energy density on the level of the residual stresses induced [25,26].

Experimental work also shows how manufacturing conditions influence the formation of residual stresses in components. Buchbinder [27] determined through an investigation of aluminum cantilever specimens that a build plate preheating of 200 °C significantly decreases residual stresses. The development of residual stresses in the depth direction shows that there are tensile residual stresses on the surface, compressive residual stresses inside the component and tensile residual stresses again in the lower areas [2,28].

For process related multi pass welds, Radaj [29] also describes an uneven distribution of residual stresses. The last welded layer has high tensile residual stresses, whereas the residual stresses in the over-welded layers are relieved by the subsequent heat input.

The following work investigates whether the described relationships are confirmed, how residual stresses and distortion depend on the process parameters and influence each other. Therefore, a dependence of residual stresses and deformations on the investigated process parameters shall be shown first. Additionally, it is expected that the cantilever deformation correlates with the residual stress level, which means large residual stresses are accompanied by large deformations.

## 2. Materials and Methods

In order to gain a better understanding of the effects leading to residual stresses and distortions in additively manufactured metal parts this paper presents numerical investigations of the laser powder bed fusion process of austenitic steel 316L. Therefore, a detailed finite element analysis is performed that takes into account the physical effects identified as relevant for the formation of temperature induced residual stresses. Due to the limited size of the model, a high resolution of heat source, scanning strategy and individual layers is possible. At the same time, the geometry allows an investigation of both residual stress and distortion, since it is based on the well-known cantilever geometry. This allows for a comprehensive evaluation of mechanisms for the development of residual stresses and an investigation of the interaction between individual melt paths and layers. In addition, the mutual dependence of deformation and residual stresses can also be investigated. The novelty of this work is the geometry used and the possibility to consider distortion and residual stress in one model. This is made possible by simulating multiple layers while still maintaining appropriate resolution of the heat source and individual melt paths. The approach combines proven methods for a parametric investigation of the interaction of process parameters and process result, which is new in this form.

The following paragraphs describe the finite element model of the LPBF manufacturing process, the simulation approach used, as well as boundary conditions, heat source and material definition.

### 2.1. Approach

**Figure 2:**
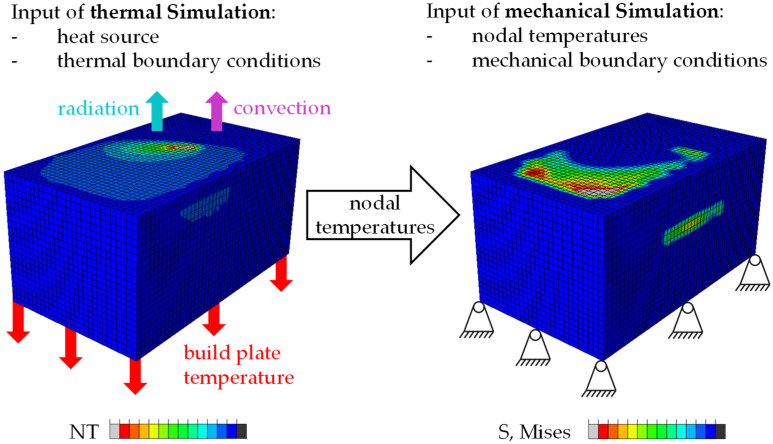
Approach used: weakly-coupled thermomechanical simulation with boundary conditions.

All simulations done in this study use a weakly-coupled thermomechanical analysis approach. Figure 2 shows the two geometrically identical models also requiring identical meshing and gives a schematic overview of the boundary conditions. First, a time-varying temperature field is calculated in an implicit heat transfer analysis using thermal boundary conditions and a volumetric heat source described below. In a subsequent implicit mechanical calculation, Abaqus reads in this temperature field and calculates thermal expansions and shrinkages considering temperature-dependent material properties. Finally, this mechanical simulation calculates stresses, strains and displacements induced by the manufacturing process.

### 2.2. Geometry

**Figure 3:**
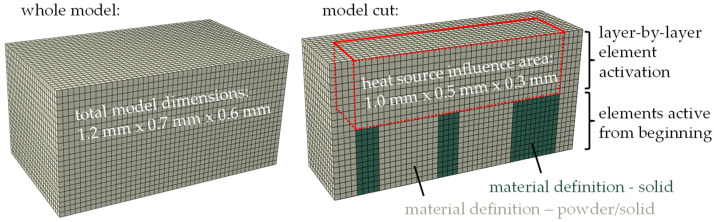
Finite element model—total dimensions and model cut with influence area of heat source.

In order to enable a simulation with high temporal and spatial resolution, the manufacturing of a small cantilever in the size of only a few millimeters is modeled. Figure 3 shows the complete model with total dimensions of 1.2 mm × 0.7 mm × 0.6 mm and an amount of 29,568 elements. Elements of type DC3D8 (thermal model) and C3D8 (mechanical model) are used in the simulations.

In the upper area, the additive manufacturing of 5 layers (60 µm) or 10 layers (30 µm) can be simulated using a layer-by-layer element activation. The influence area of the applied heat source, also representing the manufactured area is highlighted in Figure 3 with dimension of 1.0 mm × 0.5 mm × 0.3 mm. A moving heat source is applied on each layer, enabling the use of different scanning strategies. Below, solid support structures are modeled as a starting point for the additive manufacturing. A detailed description of the heat source, the layer-by-layer element activation and the different material states follows.

### 2.3. Boundary Conditions

Figure 2 schematically shows the applied boundary conditions during the simulation. In the thermal analysis, heat loss to the build chamber is always applied to the top active element layer. An Abaqus film condition with film coefficient of 20.0 W/(m^2^*K) (assumption based on [30] (p. 646)) and the Abaqus radiate command with emissivity of 0.4 (assumption based on [31]) together with the definition of an ambient temperature enable the simulation of thermal conduction and radiation. The ambient temperature for film and radiate is always identical and depends on the modeled preheating. Additionally, for the simulation of a build plate heating, all nodes of the bottom surface have a defined preset temperature in the thermal analysis. Moreover, the assignment of an initial temperature models preheating of the powder bed and the machine. In the following mechanical analysis, all nodes of the bottom surface are fixed against movement in z-direction and rotation to account for a continuing, stiff build plate. Nodal movement in x and y direction are partially permitted in the mechanical simulation to prevent unphysical tensions during cooling processes. The model accounts for cooling processes by adjusting the boundary conditions from the original process conditions to room temperature. Changes of the bottom temperature are ramped over time during the simulation step.

### 2.4. Layer-by-Layer Manufacturing

In the additive manufacturing process, a repeated layer application of new powder takes place. To simulate this effect, the model uses an Abaqus model change command first deactivating all upper element layers and successively reactivating them. Elements are not considered physically in the simulation until they are activated.

Another application of the Abaqus model change command is the partial separation of the cantilever from the build plate at the end of the simulation. For this purpose, predefined elements are deleted in the lower area of the support structures, which corresponds to the simulation of an eroding or saw cut.

### 2.5. Material

**Figure 4:**
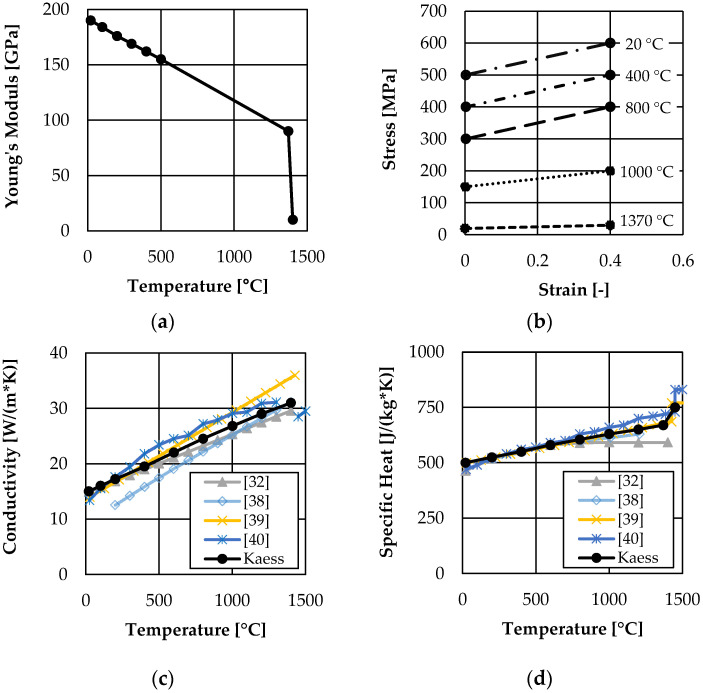

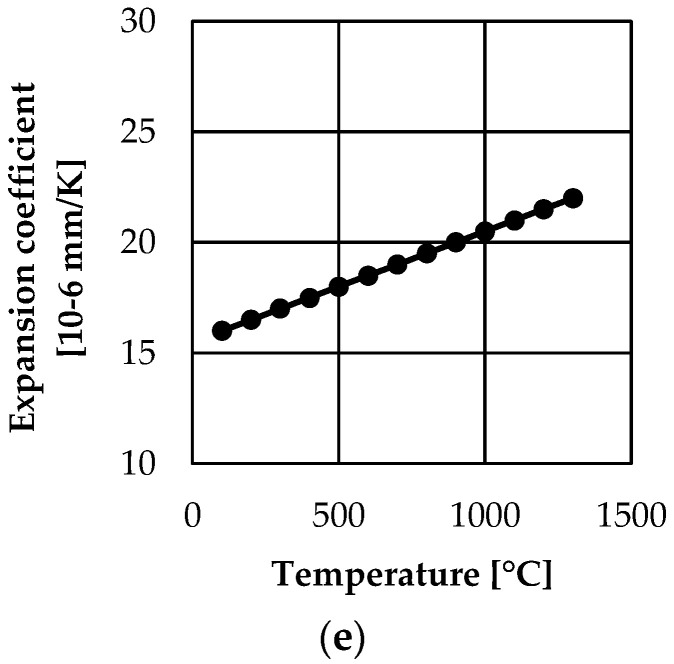
Temperature dependent material properties of 316L: (**a**) Young’s Modulus (value at room temperature [33] vertical; slope from 100 °C to 500 °C [36,37]; values at melting temperature: assumption) (**b**) Flow curve (values at room temperature [33] vertical—rounded; values at higher temperatures: assumption) (**c**) Thermal conductivity (derived from [32,37,38,39,40]) (**d**) Specific heat (derived from [32,37,38,39,40]) (**e**) Thermal expansion (values up to 500 °C [36,37]; values from 500 °C: slope assumed according to [39]).

Since the development of residual stresses strongly depends on the mechanical and thermal behavior of the material, properties in the complete temperature range of the manufacturing process are to be considered. Therefore, the simulation uses temperature-dependent material properties of 316L as well as a user subroutine for switching from powder properties to solid behavior during the modeled manufacturing process. Figure 3 shows two areas with different initial conditions of the material. Green areas highlight support structures initially modeled as solids so their production does not have to be simulated. The following layers and the surrounding powder material have a material definition, which initially uses powder properties and allows for switching to solid (gray areas in Figure 3). The change of material properties from powder before melting to solid after re-solidifying is modeled using a field variable in Abaqus. By default, all elements representing one layer are activated with powder properties assigned by a field variable FV = 0. During the simulation, a user subroutine USDFLD is used to permanently check the temperature of each element and switch the field variable to FV = 1 if the melting temperature of the material is reached. An element switched to FV = 1 remains solid for the rest of the simulation. The very low mechanical properties of molten material in the liquid state are taken into account by a temperature-dependent material definition of solid. For example, yield strength reduces to a numerically realizable minimum if the solid material reaches melting temperature. Table 1 and Figure 4 show the properties of the solid (and molten) material used in the simulation. For this work, the literature values are averaged and partially rounded. Abaqus interpolates linearly in the given ranges and keeps the properties constant for lower and higher values.

**Table 1:** Parameters for modeling 316L.

Latent heat [32]	280	kJ/kg
Melting interval [33,34]	1370–1400	°C
Density [33,35]	8.0	g/cm^3^

The metal powder used as raw material for additive manufacturing is a bulk of spherical particles filled with gas in between. However, to gain a feasible solution, the structural mechanics finite element model approach only allows for a powder description as a continuum like a solid. Consequently, the simulation can use reduced properties to model the powder behavior as an integral value. Considered as continuum, some physical and mechanical properties of powder are partly the same, others vary compared to the solid material. Accordingly, the definition of specific heat, expansion coefficient, latent heat and melting interval corresponds exactly to the temperature-dependent values for the solid 316L shown above. Table 2 shows further properties for modeling the powder. The values of Young’s Modulus and Yield Stress represent numerically realizable minimal mechanical properties.

### 2.6. Heat Source

The laser beam, selectively melting the powder material, is modeled by a hemispherical volumetric heat source with Gaussian intensity distribution. This kind of heat source is described by Goldak et al. in [17]. Equation (1) shows how the power density *q* [mW/mm^3^] is calculated using a power *P* [mW], a laser absorption *η* [-] and a laser beam radius *r* [mm]. Laser absorption *η* = 0.5 is kept constant in all calculations in this work [20,31,32].

(1) $$q\left(x,y,z\right)=\frac{6\;\sqrt{3}\;P\;\eta \;}{r^{3}\pi \;\sqrt{\pi }}\exp\left[-3\frac{x^{2}+y^{2}+z²}{r²}\right]$$

**Figure 5:**
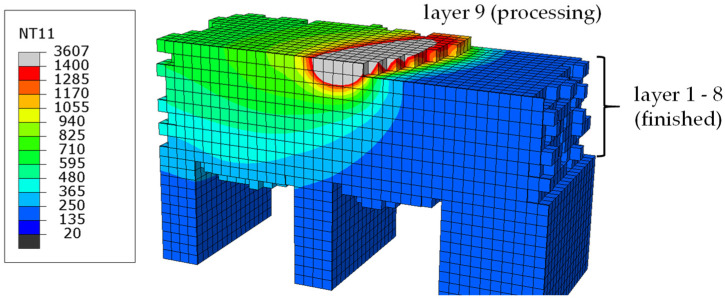
Temperature distribution [°C] around hemispherical heat source—only elements in solid/liquid state are displayed.

In the model, the Abaqus user subroutine DFLUX uses Equation (1) to implement this kind of heat source on the manufactured area. A heat source motion and modeling of different scanning patterns is realized by time-dependent modification of coordinate variables *x*, *y*, *z* in Equation (1). Figure 5 shows the temperature distribution around the heat source in a model cut. In Figure 5, all elements in powder state are hidden, to illustrate the temperature-dependent switch from powder to solid material.

### 2.7. Scanning Strategy

**Figure 6:**
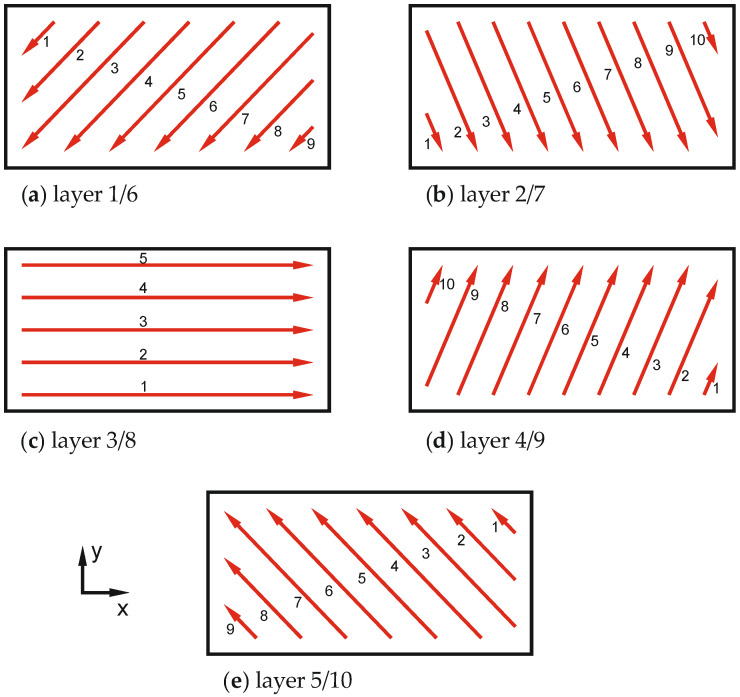
Scanning strategy—67° rotation.

In addition to the description of the heat source, the subroutine DFLUX also contains a definition of the scanning strategy for the model. By moving the heat source described above on the top layer of the model, a movement of the laser and thus the layer-by-layer exposure of the surface is simulated. In this study, a linear scanning strategy with an incremental rotation of 67° is always used, as shown in Figure 6. A linear scanning strategy is chosen because the currently established strategies are usually based on linear patterns and their locally different arrangement. A rotation of 67° is intended to contribute to the most isotropic conditions possible. This type of rotation is also used in [43,44,45]. For a layer thickness of 60 µm, the production of five layers is modeled, so that the five patterns shown in Figure 6 are applied one after the other. For a layer thickness of 30 µm, the model has 10 layers, so the scanning strategy shown is repeated from layer six. Figure 6 also contains a numbering of the scan paths, which corresponds to the order of exposure within a plane.

### 2.8. Parameter Study—Modeled Manufacturing Parameters

In order to evaluate the influence of different manufacturing conditions on the residual stress distribution, numerous different parameter sets are used in the simulations. Table 3 shows an overview of these parameters. A standard parameter configuration, highlighted in bold, is used as a reference. Based on these standard parameters layer thickness, build plate temperature, laser power and speed are varied separately according to Table 3.

For example, the investigations on layer thickness and preheating always use constant laser parameters (250 W, 850 mm/s). Whereas, the variation of the laser parameters is made with constant preheating (150 °C) and layer thickness (30 µm). The variation of the laser power is carried out once with constant scanning speed (850 mm/s) and once with varying scanning speed to achieve a constant line energy density (LED = 0.29 J/mm). Laser spot size and hatch distance are always constant. The parameters used in each case can be found in the diagrams showing the results.

### 2.9. Simplifications

The model focuses on the most important effects with regard to the objective of this study, which is the modeling of residual stresses and distortion in 316L material. However, the finite element simulation approach used does not allow a reasonable representation of all physical effects in the process. For example, neither individual powder particles nor melt bath dynamics, such as the Marangoni effect, are represented in the model. In contrast, a liquid flow in the melt pool is simulated by [18,19,32], but only a limited model size and few scan lines are considered here. The aim of these investigations is primarily the formation of the melt pool. However, these effects are considered less relevant for the formation of distortion and residual stresses. Furthermore, the model does not make any predictions regarding the microstructure and possible microstructural transformations. For the present material, austenitic steel 316L, no residual stress and distortion influencing effect is to be expected here either, so that a consideration of the microstructure is not necessary.

## 3. Results

**Figure 7:**
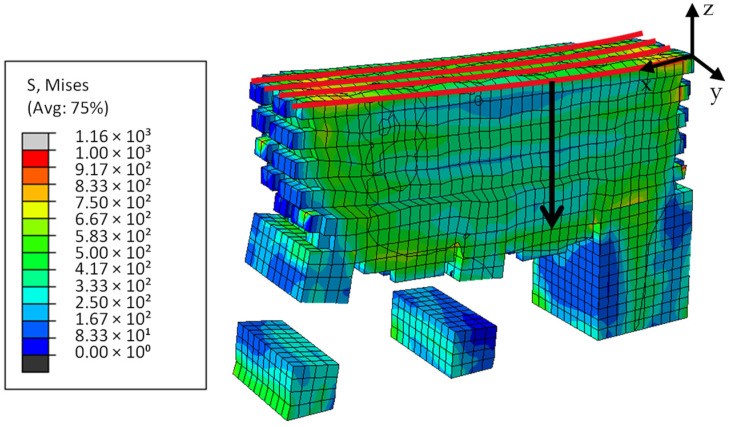
Model cut: cantilever after partial separation—path for distortion evaluation marked in red and path for stress evaluation marked in black.

The residual stress evaluation of the models takes place after simulation of the additive manufacturing process and before a partial cantilever separation. The black arrow in Figure 7 shows the predefined path in negative z-direction used for this purpose. In the simulation, the partial separation and an evaluation of the resulting cantilever bending follows. This bending is evaluated along the cantilever surface shown by parallel red lines in Figure 7.

During the simulations, some convergence issues due to large nonlinearities occurred. To reduce these difficulties and to improve convergence a line search algorithm was used in Abaqus.

**Figure 8:**
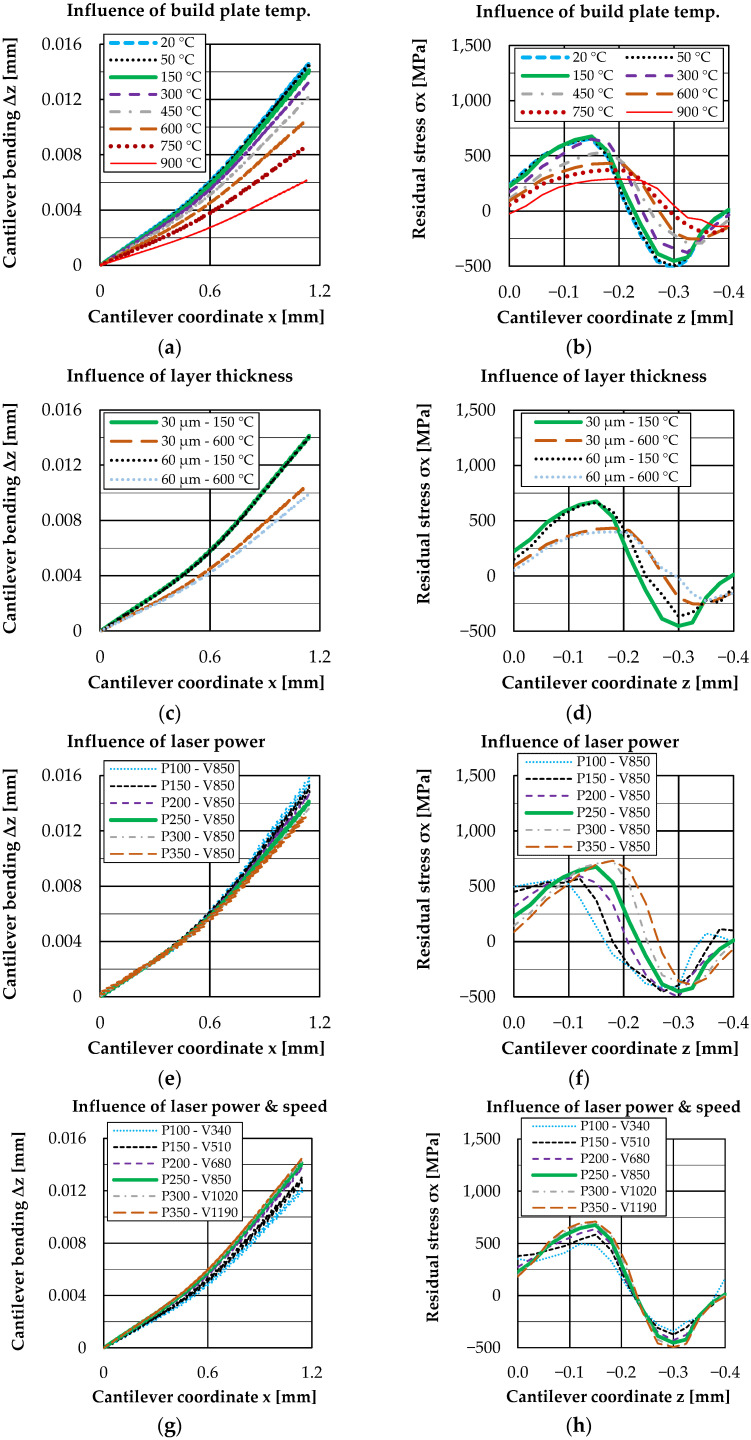
Results: Bending (**a**) and residual stress (**b**) for a variation of build plate temperature; bending (**c**) and residual stress (**d**) for a variation of layer thickness; bending (**e**) and residual stress (**f**) for a variation of laser power; bending (**g**) and residual stress (**h**) for a variation of laser power & speed.

**Figure 9:**
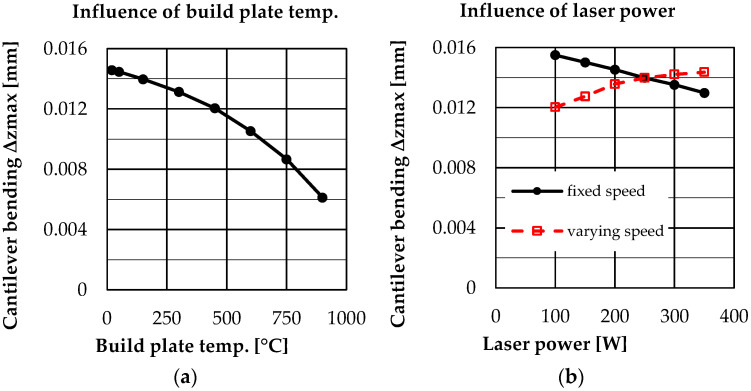
Maximum bending at cantilever front position dependent on build plate temperature (**a**) and laser power (**b**)—fixed speed brings variation in LED—varying speed keeps LED constant.

Figure 8 shows the results for a variation of build plate temperature, layer thickness, laser power and laser power & speed. The bending line along the longitudinal axis of the cantilever beam x is shown on the left and the residual stress curve over the cantilever depth z is shown on the right. For a quantitative comparison, Figure 9 additionally shows the maximum deflection at the cantilever front position.

The evaluation of the results (left figures) always shows an upward cantilever deflection of varying degree, which depends on the different process parameters used. In the right figures, the residual stresses principally show an initially increasing tensile range at the top of the cantilever, which changes to a compressive range at the bottom. A variation of the process parameters leads on the one hand to a change of the maximum and minimum stress values and on the other hand to a shift of the curve in depth direction.

Looking at the influence of the build plate temperature (Figure 8a,b), it can be seen that bending significantly reduces from 300 °C. The results show that an increasing build plate temperature also leads to a reduced development of residual stresses in the tensile area as well as in the compression area. Here the effects are clearly visible from a temperature of 450 °C onwards.

For the analysis of the influence of layer thickness, the Figure 8c,d show representative results at 150 °C and 600 °C. Here it can be seen that both the bending line and the residual stress curve of 30 µm and 60 µm layer thickness look very similar for 150 °C and only slightly different for 600 °C. It should be noted that, except for the layer thickness, the two variants were simulated with identical production parameters. This means that the volume energy density is not constant but differs by a factor of two due to the different thickness. Thus, it is even more remarkable that the results are so similar.

The variation of the laser power (Figure 8e,f) shows that the cantilever deflection decreases with increasing laser power (laser speed constant, increasing line energy density). This behavior is rather unexpected, since the maximum values of the residual stresses also increase with increasing laser power. However, in addition to the change in the absolute maximum values of the residual stresses, there is also a shift of the residual stress curve in the depth direction. For a low laser power, the maximum of the residual stresses occurs geometrically higher in the cantilever than for a high laser power. This shift in the residual stress curve seems to be decisive for the deflection of the cantilever.

If, in addition to the laser power, the laser speed is also varied in order to keep the line energy density (LED) constant, a different situation emerges. This is shown in Figure 8g,h. The cantilever deflection increases with increasing laser power (laser speed varied, LED constant). Here, the maximum values of the residual stresses increase with increasing power, as seen before for only varying the laser power. The difference occurs in the unchanged position of the depth profile of the residual stresses. This has the effect that the deflection changes qualitatively to the same extent as the maximum values of the residual stresses for a constant LED.

As described above, Buchbinder [27] found a decrease in cantilever deflection with experimental increase of preheating. This correlation is also shown by the simulated results in this work, so that it can be concluded that the simulations qualitatively represent the correct behavior. Buchbinder found that distortion no longer occurs in aluminum specimens above 250 °C preheating. Future experiments at 316L should verify whether this effect can also be expected in 316L at higher temperatures, in order to further develop the model accordingly. Mercelis [2] and Wu [28] describe a residual stress profile with tensile stresses in the near surface region and compressive stresses in geometrically deeper regions. This can also be seen qualitatively in the simulation results. Furthermore, the results are consistent within this work and the parameter study carried out, allowing a comparison between the parameters used.

Further comprehensive investigations of the predicted behavior with different experimental methods are planned. However, it should also be noted here that the experimental determination of residual stresses is associated with great uncertainties as well as variations between different methods. For this reason, a numerical investigation of the residual stresses is also important in order to pre-select suitable study parameters and compare the plausibility of future results.

The results obtained so far show very different and partly complementary influences of the process parameters on residual stresses and distortion. To deeper investigate the influences of laser power further simulations were conducted with a laser power variation from layer to layer.

The aim is to determine the extent to which deeper located layers and near-surface layers influence the result of cantilever bending and residual stresses in each case. For scanning strategy and all other parameters, the default configuration is used. Table 4 shows how the laser power is adjusted from layer to layer.

**Figure 10:**
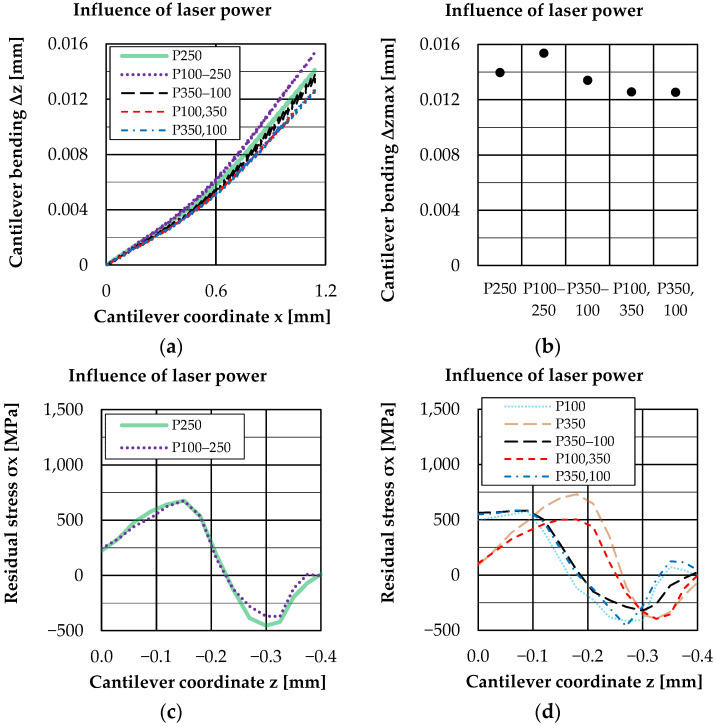
Cantilever bending (**a**,**b**) and residual stress (**c**,**d**) for laser power variation from layer to layer.

Figure 10 shows the influence of these layer wise power variations on the cantilever bending line and total bending at the cantilever front position. Figure 10 also contains an illustration of residual stress curves for these variations in comparison to a constant laser power already shown before.

For a clear presentation, the residual stresses are divided into two graphs. Here, a comparison with constant laser power shows that the laser power used in the top layers is decisive for the course of the residual stresses. The power used in deeper layers seems to have less influence on the residual stress curve. The evaluation of the bending line shows a less clear result, so that the derivation of such relationships is not possible here. These results, as well as the previous results with constant laser power, suggest that the residual stress profile is influenced by the power and line energy density, especially of the upper layers. It can be concluded that the weld penetration depth, which depends on the laser power, influences the position of the residual stress profile and thus also the distortion.

## 4. Discussion

The evaluation of the results shows that a variation of the process parameters can have very different and partly complementary effects on distortion and residual stresses. A change in the build plate temperature shows a clear tendency in stress and distortion. At 300°C and above, the first effects of avoiding distortion become apparent. From 450°C, these become more pronounced and the residual stresses also decrease significantly. In contrast, the processes become more complex as soon as the laser parameters are changed, affecting energy input and melt pool geometry. Here the cantilever deflection does not always provide the complete picture for evaluating the residual stresses in the component, since both the maximum values and the spatial position of the residual stress profile affect the deflection. In principle, the model predicts a strong influence of the upper layers on the formation of residual stresses. The results obtained allow the conclusion that the model developed is suitable for the preliminary investigation of process parameters in laser powder bed fusion and can predict influences on residual stresses and distortion. From the results, it could be concluded that the preheating should be as high as possible to avoid residual stresses and distortion. However, it must be taken into account that other material properties also depend on this temperature. A high preheating may result in altered strength properties and ductility. Another conclusion could be to reduce the laser power and speed in order to optimize the process in terms of residual stresses and distortion. Here it is important not to reduce the laser power and thus the energy density alone in order to ensure sufficient layer bonding and part density. However, a greatly reduced scanning speed leads to longer process times, which may not be appropriate from an economic point of view. The discussion shows that it is possible to influence residual stresses and deformation through the process parameters, but that this can only be done sensibly within certain limits. This is due to the multi-complex relationships between process parameters and the resulting material and component properties in additive manufacturing in general.

## 5. Conclusions

A detailed finite element simulation approach with user coded subroutines is developed to better understand the relevant mechanisms for the formation of residual stresses in parts being additively manufactured by laser powder bed fusion (LPBF). The numerical analyses show that a layer-by-layer manufacturing with a laser heat source from a powder raw material can be modeled. However, using a high temporal and spatial resolution is only feasible for modeling the manufacturing of a small volume. In addition, the required computational effort can only be realized for research purposes. The model allows many further investigations, such as different scanning strategies, further laser parameters and materials, in order to be able to make an optimized parameter preselection with regard to residual stresses and distortion. Additionally, the user subroutines could be extended to include microstructural transformation in order to be able to model other materials such as maraging steels. Further studies could also include a more detailed description of the liquid state of molten material to better evaluate the influences of the current simplifications. An extension of the material description to include a time-dependent behavior (creep/relaxation) could improve the accuracy of the model for investigating residual stress relief during the process and subsequent heat treatment. In the future, new numerical studies as well as a comprehensive investigation of the predicted behavior with different experimental methods are planned. For this experimental work, the simulation results obtained can be used for the pre-selection of suitable study parameters.

## Figures and Tables

**Table 2:** Parameters for modeling 316L powder.

Density [41]	4.0	g/cm^3^
Young’s Modulus [assumption]	10.0	GPa
Yield Stress [assumption]	1.0	MPa
Conductivity [11,42]	0.15 (20 °C) 0.6 (1370 °C)	W/(m*K)

**Table 3:** Modeled manufacturing parameters.

Layer thickness	**30**, 60	µm
Build plate temperature	20, **150**, 300, 450, 600, 750, 900	°C
Laser power	100, 150, 200, **250**, 300, 350	W
Laser speed	340, 510, 680, **850**, 1020, 1190	mm/s
Laser beam radius	**50**	µm
Hatch spacing	**100**	µm

**Table 4:** Laser power [W] dependent on layer 1, 2, 3, 4, 5, 6, 7, 8, 9, 10.

P100–250	100, 150, 200, 250, 250, 250, 250, 250, 250, 250
P350–100	350, 350, 300, 300, 250, 250, 150, 150, 100, 100
P100, 350	100, 100, 100, 100, 100, 350, 350, 350, 350, 350
P350, 100	350, 350, 350, 350, 350, 100, 100, 100, 100, 100

## Data Availability

The data presented in this study are available on request from the corresponding author.
